# The molecular landscape of peach allergy in Tunisia: new insights

**DOI:** 10.3389/fimmu.2026.1696481

**Published:** 2026-03-03

**Authors:** Nader Ben Nejma, Dhouha Krir, Yousr Galai, Ahlem Ben Hmid, Ines Ben Sghaier, Yosra Nasri, Wassila Abdennadher, Hayet Kbaier, Hechmi Louzir, Nissaf Bouaffif Alaya, Mélika Ben Ahmed, Imen Zamali, Samar Samoud

**Affiliations:** 1 Laboratory of Clinical Immunology, Institut Pasteur de Tunis, Tunis, Tunisia; 2 Faculty of Medicine de Tunis, University of Tunis El Manar, Tunis, Tunisia; 3 Faculty of Pharmacy of Monastir, University of Monastir, Monastir, Tunisia; 4 Laboratory of Transmission, Control and Immunobiology of Infection, Institut Pasteur de Tunis, Tunis, Tunisia; 5 National Observatory of New and Emerging Diseases, Ministry of Health, Tunis, Tunisia; 6 Faculty of Medicine of Sousse, University of Sousse, Sousse, Tunisia

**Keywords:** molecular allergen, peach allergy, sensitization profile, specific IgE, Tunisia

## Abstract

**Background:**

Peach allergy represents a significant clinical problem in Mediterranean populations, yet molecular characterization remains limited in North African countries. This study provides the first comprehensive analysis of peach sensitization patterns in Tunisia using component-resolved diagnostics.

**Objective:**

To characterize molecular sensitization profiles to peach allergen components, correlate these with clinical manifestations, and evaluate predictive biomarkers in a Tunisian cohort.

**Methods:**

A retrospective study was conducted including 63 patients referred for suspected peach allergy to the Pasteur Institute of Tunisia between March 2022 and March 2025. After application of exclusion criteria, 49 patients were included in the final analysis. Total and specific IgE levels were measured using ImmunoCAP^®^ technology. Component-resolved diagnostics targeted rPru p 1, rPru p 3, rPru p 4, and rPru p 7. Reaction severity was assessed using oFASS-3. Statistical analyses included correlation studies and ROC curve analysis.

**Results:**

Thirty-eight patients (77.5%) demonstrated peach allergy with median age 10 years [7-14]. Clinical manifestations included urticaria (86.6%), angioedema (39.4%), respiratory symptoms (rhinitis 42.1%, bronchospasm 23.6%), oral allergy syndrome (32.4%), and anaphylaxis (10.5%). Pru p 3 was predominant (84.2% of patients, median 1.215 kUA/L). Pru p 7, Pru p 1, and Pru p 4 sensitization occurred in 10.5%, 7.8%, and 2% respectively. Peach-specific IgE predicted Pru p 3 sensitization with 100% sensitivity and 66.7% specificity (cut-off 0.23 kUA/L). Strong correlation existed between peach-specific IgE and Pru p 3 levels (ρ = 0.942). No associations were identified between biomarkers and clinical severity.

**Conclusions:**

Peach allergy in Tunisia follows the Mediterranean phenotype with predominant Pru p 3 sensitization and significant clinical severity. These findings establish the molecular foundation for evidence-based diagnosis while highlighting the need for region-specific therapeutic approaches.

## 1 Introduction

Food allergy is increasingly recognized as a major global public health issue, affecting nearly 10% of the population across diverse geographic regions and age groups (1–3). Beyond the substantial economic burden, it imposes on healthcare systems, food allergy considerably reduces the quality of life of affected individuals. Within this spectrum, peach allergy (*Prunus persica*) has emerged as one of the most common fruit allergies in Mediterranean countries, including Tunisia, where it frequently represents the leading cause of fruit-induced allergic reactions (4, 5). Clinical manifestations range from mild, localized mucocutaneous symptoms to life-threatening anaphylaxis (3, 6), and cross-reactivity with other plant foods often complicates diagnosis and management (5).

Advances in molecular allergology have transformed the diagnostic approach to food allergy through component-resolved diagnostics (CRD) (1, 6). CRD supports individualized risk stratification, helping to prevent unnecessary dietary restrictions while accurately identifying patients at risk of severe reactions (3). Several clinically relevant peach allergens are recognized by the WHO/IUIS Allergen Nomenclature Sub-Committee (7), including Pru p 3 (non-specific lipid transfer protein, LTP), Pru p 7 (gibberellin-regulated protein, GRP), Pru p 1 (pathogenesis-related protein-10, PR-10), Pru p 2 (thaumatin-like protein), Pru p 4 (profilin), Pru p 9 (pathogenesis-related protein-1), and Pru p 10 (polygalacturonase). Pru p 3 (LTP) represents the major allergen in Mediterranean populations (6, 8) and the primary trigger of LTP syndrome, with thermostability and digestive resistance contributing to systemic reactions (6). Pru p 7 is recognized as an emerging major allergen in certain populations, with sensitization associated with severe anaphylactic reactions (9, 10). In contrast, Pru p 1 and Pru p 4 are thermolabile proteins associated with cross-reactivity and mild oral allergy syndrome. The clinical relevance of Pru p 2, Pru p 9, and Pru p 10 remains insufficiently established, and are not currently used in routine clinical practice. Despite rapid CRD adoption in Europe, implementation in North African countries remains limited. In Tunisia, data on the molecular epidemiology of peach allergy are virtually absent, hindering the development of region-specific diagnostic and therapeutic strategies.

This study represents the first comprehensive molecular analysis of peach sensitization in a Tunisian population. Our objectives were: (i) to characterize sensitization patterns to peach allergen components using CRD, (ii) to correlate molecular profiles with clinical manifestations, and (iii) to evaluate whether total IgE, specific IgE, major CRD and IgE ratios could serve as predictive markers of severity.

## 2 Methods

### 2.1 Study design and population

We conducted a retrospective analytical study including 49 patients assessed for suspected peach allergy at the Department of Clinical Immunology-Allergology, Pasteur Institute of Tunisia, between March 2022 and March 2025. Referrals were received from various specialists, including pulmonologist-allergist, and pediatricians covering the different region of Tunisia.

A standardized form was used to systematically retrieve clinical data from the medical records of patients with allergic reactions related to peach exposure. Reaction severity was assessed using the Oral Food Allergy Severity Score (oFASS-3), a classification system proposed by *Muraro* et al. and endorsed by the European Academy of Allergy and Clinical Immunology (11).

Both skin prick tests (SPTs) with commercial extracts and prick-to-prick tests with fresh peach and other relevant allergens were performed when clinically appropriate and considered safe, following standard protocols. However, these tests were not systematically performed in all patients, as many physicians in private practice tend to avoid them. The two procedures differ in that SPTs with commercial extracts use standardized allergen concentrations, whereas prick-to-prick tests with fresh allergens assess patient-specific reactivity to native food proteins.

Oral food challenge (OFC), recognized as the gold standard for confirming food allergy, was not performed in any patient due to safety concerns in children with prior reactions and constraints inherent to real-world clinical practice. Diagnosis of peach allergy relied on a combination of detailed clinical history, documentation of allergic reactions, and serological assessment, followed by CRD when indicated.

Patients were excluded if key clinical data were missing—mostly due to loss to follow-up—or if SPT results were incomplete. The latter included cases in which patients consulted practitioners not qualified to perform SPT, when the test was unavailable at the time of consultation, contraindicated because of a high risk of severe reaction, declined by the patient, or rendered uninterpretable due to technical issues. In total, 14 patients were excluded for these reasons.

### 2.2 IgE measurements

Venous blood samples were collected in plain tubes, centrifuged at room temperature, and sera aliquoted and stored at –20°C until analysis. Total serum IgE (tIgE) levels were quantified by turbidimetry (Optilite™ analyzer, The Binding Site^®^, Birmingham, UK) and interpreted according to age-adjusted reference values. Specific IgE (sIgE) to whole peach extract (f95) was measured using ImmunoCAP^®^ (Phadia™ 200 systems, ThermoFisher Scientific^®^, Uppsala, Sweden), and when positive, sIgE to molecular components was subsequently assessed. CRD were performed when indicated, targeting recombinant allergens: rPru p 3 (nsLTP, f420), rPru p 7 (GRP, f454), rPru p 1 (PR-10, f419) and rPru p 4 (profilin, f421).

**Figure 1:**
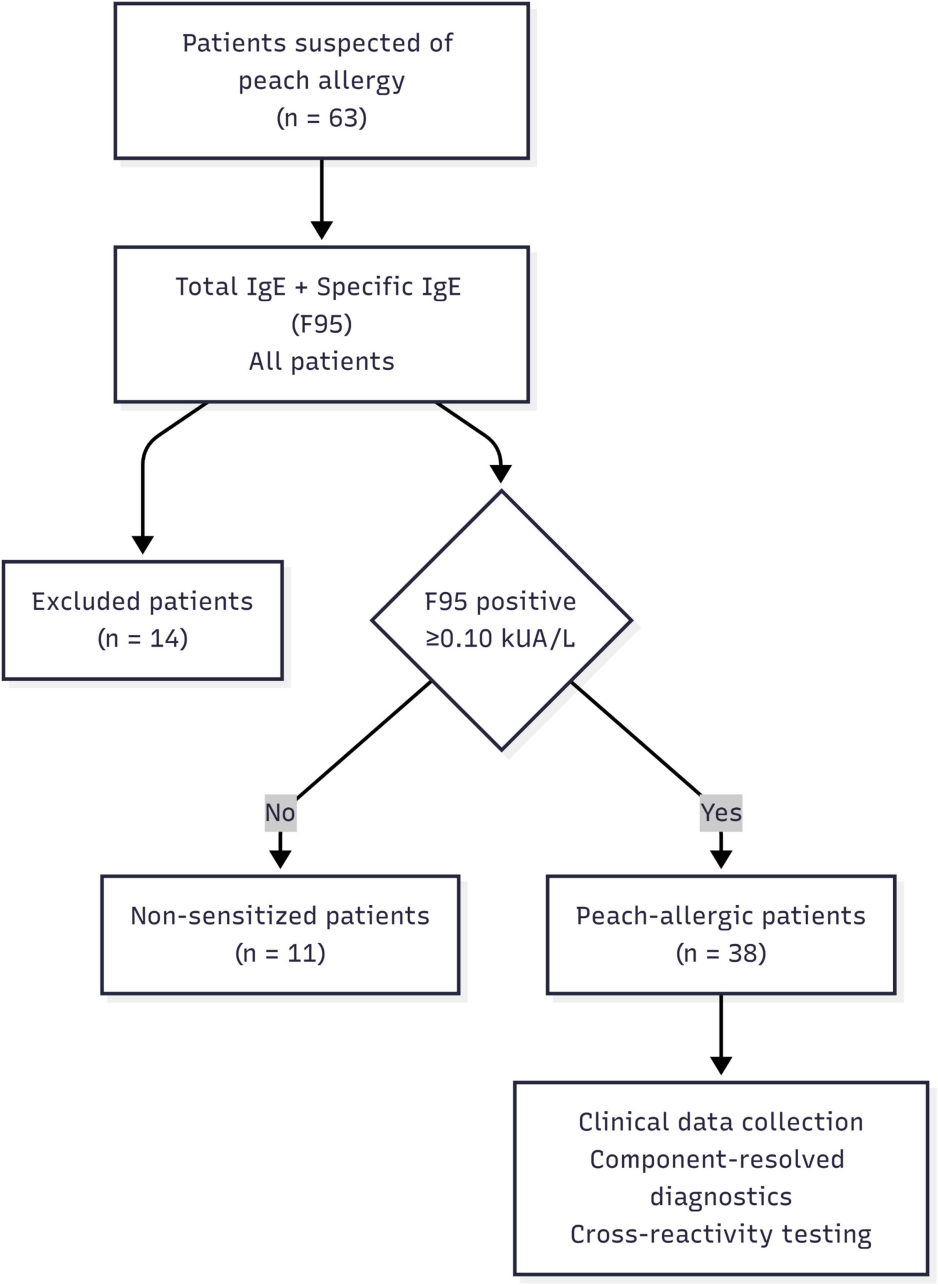
Flowchart depicting participant inclusion in the peach-sensitized group. * Low-level sIgE sensitization (0.1–0.35 kUA/L) was interpreted with caution and not systematically equated with clinical allergy.

For analytical purposes, a cut-off value of 0.1 kUA/L was used to define sIgE sensitization, corresponding to the analytical detection limit of the ImmunoCAP^®^ assay and in line with published literature (12, 13). However, for descriptive and comparative analyses of clinical peach allergy, sIgE values ≥0.35 kUA/L were interpreted with greater caution and considered more strongly suggestive of clinically relevant allergy, in accordance with current consensus. The assay has an upper limit of quantification (ULOQ) of 100 kUA/L. Peach allergy was suspected when sIgE to f95 was ≥0.10 kUA/L, with clinical symptoms and, when available, SPTs supporting the diagnosis, which systematically led to further molecular testing. The algorithm summarizing this approach is illustrated in **Figure 1**.

Cross-reactivity was assessed, when clinically indicated, by sIgE to Rosaceae fruits (apple, apricot, pear), tree nuts (hazelnut, walnut, almond, pistachio), pollens (olive, cypress, birch) and house dust mites (HDMs) (*Dermatophagoides pteronyssinus*, *Dermatophagoides farinae*).

### 2.3 Statistical analysis

Statistical analyses were performed using SPSS software, version 11 (IBM Corp., Armonk, NY, USA) and GraphPad Prism 5 (Dotmatics^®^). Continuous variables were expressed as medians with interquartile ranges [Q1–Q3] or as means ± standard deviation (SD), depending on the distribution. Categorical variables were summarized as frequencies and percentages. Comparisons of categorical variables were performed with the Chi-square (χ²) test, or with Fisher’s exact test when required. Differences in medians between independent groups were assessed using nonparametric tests for non-normally distributed data. Associations between quantitative variables were evaluated using Pearson’s (*r*) or Spearman’s correlation coefficients (ρ), as appropriate. The effect size for Pearson’s correlation was estimated using the coefficient of determination (R²). Multivariable analysis to determine factors associated with CRD was performed using multiple linear regression. Variables reaching a *p*-value <0.20 in univariate testing were retained in the multivariable model. A two-tailed *p* < 0.05 was considered statistically significant. Receiver-operating characteristic (ROC) curve analysis was conducted to assess the predictive performance of various serological parameters for peach sensitization and CRD positivity. Sensitivity, specificity, and area under the curve (AUC) were evaluated, with optimal cut-off points selected based on the Youden index.

For severity prediction, only confirmed peach-sensitized patients were included in the analysis. Based on oFASS-3, reactions were dichotomized into mild-to-moderate (grades 1–2) *versus* severe (grade 3).

### 2.4 Ethical considerations

The study was approved by the Ethics Committee of the Faculty of Medicine of Sousse (CEFMSo_0002_2025). Informed consent was obtained from all patients or their guardians, and data were anonymized prior to analysis in compliance with confidentiality standards.

## 3 Results

### 3.1 Study population and basic characteristics

Sixty-three patients with suspected peach allergy were initially enrolled and underwent screening. After application of the exclusion criteria, 49 patients were included in the study for further analyses. The overall study population had a median age of 11 years [7–18] and included 22 males and 27 females, corresponding to a male-to-female ratio of 0.81. Pediatric patients (<18 years) represented 65.3% of the study population (*n* = 32).

### 3.2 Clinical features of peach-sensitized patients

Thirty-eight patients (77.5%) tested positive for sIgE to peach extract and exhibited clinical features consistent with peach allergy, forming the main population for further analyses. In this group, the median age was 10 years [7–14], with 21 males and 17 females (sex ratio = 1.23). Compared with patients negative for sIgE to f95, f95-positive patients were significantly younger (*p* = 0.021). A statistically significant association was found between sex and peach sensitization (*p* = 0.013). Detailed clinical data were available for all 38 confirmed cases. A family history of atopy was reported in 23 patients (60.5%). The median age at first documented allergic reaction was 5.5 years [4 – 8]. Whole peach was identified as the offending food in 35 patients (92.1%), while peach juice was implicated in 3 cases (7.9%). Symptoms developed immediately after ingestion in 20 patients (52.6%) and within a few minutes in 18 patients (47.4%) (**Table 1**).

**Table 1:** Demographic and clinical characteristics of patients according to sensitization, age group, and reaction severity.

Study population (n)	Age (years) (median [range])	Sex ratio (M/F)	Age at first reaction (median [range])
All patients (49)	11 [7–18]	0.81	–
f95-positive patients (38)	10 [7–14]	1.23	–
f95-negative patients (11)	32 [9.5–43]	0.1	–
*P*	0.021	0.013	–
Pediatric patients (32)	8.5 [6–11]	1.125	–
Adult patients (17)	32 [19.5–43]	0.36	–
*P*	–	0.123	–
Grade 1 reactions group (2)	17 [9–25]	0	7.5 [6–9]
Grade 2 reactions group (19)	11 [7–16.5]	1.11	5 [2.5–8.5]
Grade 3 reactions group (17)	10 [7–12]	1.83	6 [4–8]
*p*	0.466	0.208	0.496

The clinical spectrum of reactions was heterogeneous. Cutaneous manifestations were predominant, with urticaria reported in 86.6% and angioedema in 39.4%. Respiratory manifestations occurred in 52.6% of patients, with rhinitis (39.4%) and bronchospasm (23.6%) being the most frequent symptoms. Oral allergy syndrome (OAS) was observed in 32.4%, and anaphylactic shock occurred in 10.5% of cases. Complete avoidance of peach was reported by 97.3% of patients. Hospitalization was required in 21% of cases, all of whom had severe systemic reactions (**Table 2**).

**Table 2:** Clinical manifestations among peach-allergic patients, categorized by age group and reaction severity.

Peach allergy reactions *n* (%)	Peach sensitized patients	Peach sensitized pediatric patients	Peach sensitized adult patients	Grade 1 reactions group	Grade 2 reactions group	Grade 3 reactions group
	(*n*=38)	(*n*=30)	(*n*=8)	(*n*=2)	(*n*=19)	(*n*=17)
Urticaria	33 (86.8%)	28 (93.3%)	5 (62.5%)	0	19 (100%)	14 (82.3%)
Oral syndrome	13 (34.2%)	10 (33.3%)	3 (37.5%)	2 (100%)	3 (15.7%)	8 (47%)
Isolated oral syndrome	2 (5.2%)	1 (3.3%)	1 (12.5%)	2 (100%)	0	0
Quincke’s edema/Angioedema	15 (39.4%)	14 (46.6%)	1 (12.5%)	0	0	15(88.2%)
Vomiting	4 (10.5%)	3 (10%)	1 (12.5%)	0	1 (5.2%)	3 (17.6%)
Cough	6 (15.7%)	4 (13.3%)	2 (25%)	0	3 (15.7%)	3 (17.6%)
Rhinitis	15 (39.4%)	10 (33.3%)	5 (62.5%)	1 (50%)	6 (31.5%)	8 (47%)
Bronchospasm	9 (23.6%)	8 (26.6%)	1 (12.5%)	0	0	9 (52.9%)
Grade 4 anaphylaxis	4 (10.5%)	4 (13.3%)	0	0	0	4 (23.5%)

### 3.3 IgE profile analysis and diagnostic performance evaluation

**Figure 2:**
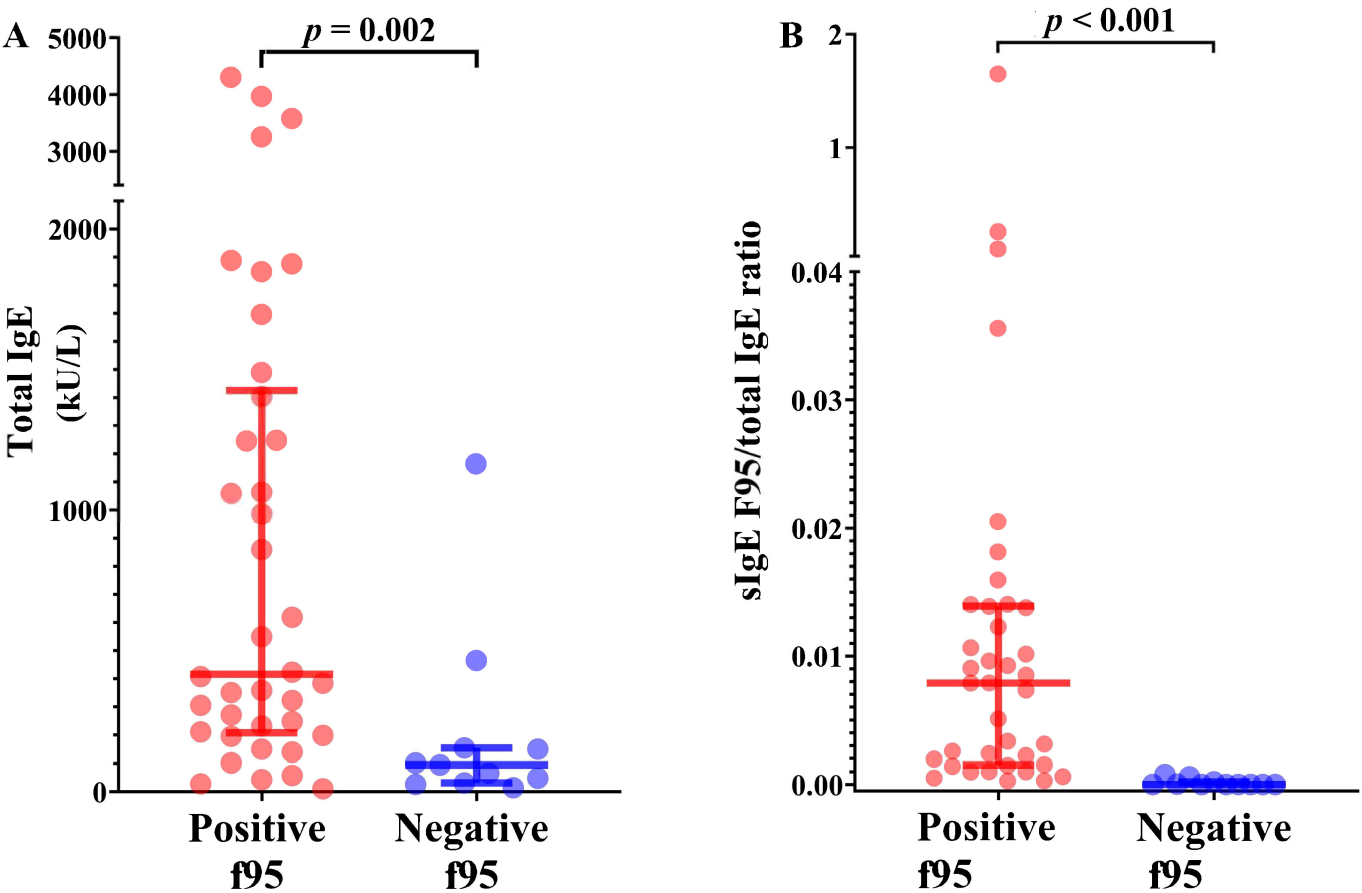
Distribution of total IgE and specific IgE/total IgE ratio according to peach sensitization status. Both total IgE levels **(A)** and the specific IgE/total IgE ratio **(B)** were significantly higher in patients sensitized to peach (Positive F95) compared with non-sensitized individuals (p < 0.05).

**Figure 3:**
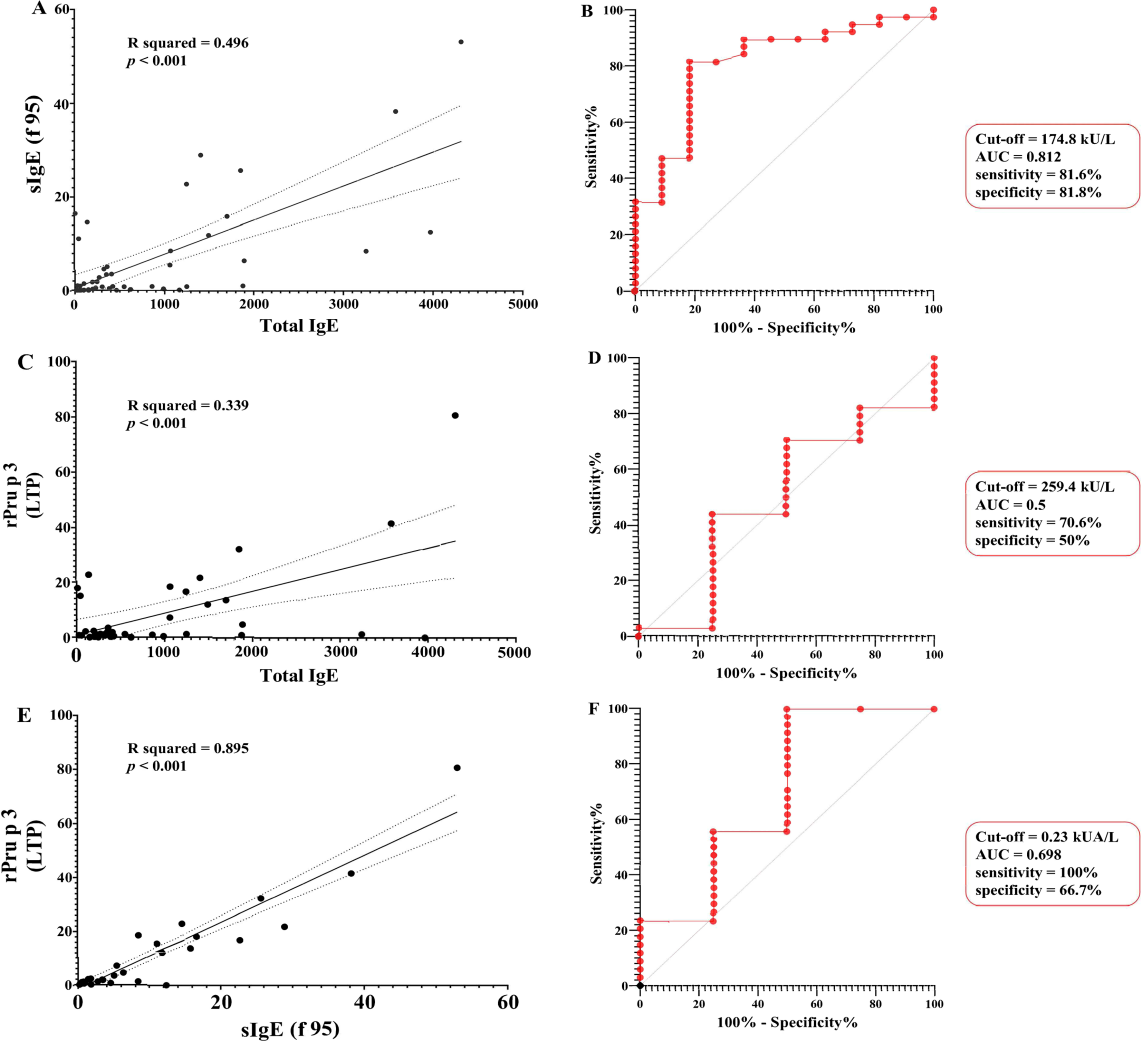
Correlation and receiver operating characteristic (ROC) analysis of IgE biomarkers for peach allergy prediction. Correlations are shown in **(A, C, E)** with corresponding ROC analyses in **(B, D, F)**. **(A, B)** Total IgE and peach-specific IgE (F95) for peach sensitization. **(C, D)** Total IgE and rPru p 3 for rPru p 3 positivity. **(E, F)** Peach-specific IgE (F95) and rPru p 3 for rPru p 3 sensitization."

tIgE levels were measured in all 49 participants. The median concentration for the entire group was 350.7 [139.9–1165.4] kU/L, with values ranging from 10 to 4306.3 kU/L. Total IgE levels were above the normal range in 94.7% of patients positive for sIgE to f95. Peach-allergic patients exhibited significantly higher tIgE levels compared with non-sensitized patients (*p* = 0.002) (**Figure 2A**). Among peach-allergic patients, the median sIgE level to f95 was 3.06 [0.77–11.74] kUA/L. No statically significant age-related differences were observed (*p* = 0.562). tIgE strongly correlated with sIgE to f95 (ρ = 0.534, *p* < 0.001), and linear regression confirmed tIgE as a significant predictor (R² = 0.496, *p* < 0.001). ROC analysis showed excellent discrimination (AUC = 0.812), with an optimal cut-off of 174.8 kUA/L in symptomatic patients, yielding 81.6% sensitivity and 81.8% specificity for f95 positivity (**Figures 3A, B**). The sIgE/total IgE ratio was significantly higher in patients with positive sIgE compared to those with negative sIgE (*p* < 0.001), while Spearman’s correlation did not reveal a significant monotonic association (ρ = 0.125, *p* = 0.455) (**Figure 2B**).

### 3.4 Sensitization profiles and component-resolved diagnostics

CRD confirmed that peach allergy is primarily driven by sensitization to rPru p 3 (LTP), the major allergen of peach. Indeed, IgE from 84.2% (*n* = 32) of patients bound to rPru p 3, with median levels of 1.215 [0.642–13] kUA/L. Conversely, IgE from 10.5% (*n* = 4) of patients bound to the newly identified rPru p 7, including one case of isolated reactivity. IgE binding to rPru p 4 (profilin) was detected in 2% (*n* = 1) of patients, while IgE from 7.8% (*n* = 3) of patients bound to rPru p 1 (PR-10 protein).

Single component sensitization was observed in 33 of 38 patients (86.8%) (**Table 3**), while the remaining 5 patients (13.2%) demonstrated co-sensitization to multiple peach components: 3 patients showed dual positivity to rPru p 3 and rPru p 7, 1 patient demonstrated concomitant rPru p 3 and rPru p 1 positivity, and 1 patient exhibited concurrent rPru p 7 and rPru p 4 sensitizations. Among the 3 patients positive for rPru p 1, 2 showed isolated PR-10 protein sensitization (**Table 4**). Similarly, 1 of the 4 rPru p 7-positive patients exhibited isolated gibberellin-regulated protein sensitization.

**Table 3:** Baseline characteristics and allergen sensitization among f95-positive peach patients (*n* = 38).

Variable	f95-positive patients (*n* = 38)
Patient characteristics	
Pediatric/adult, n	30/8
Age, years, median [range]	10 [7–14]
Sex, n (male/female)	21/17
Family history of atopy, n (%)	23 (60.5)
Skin testing	
SPT and/or prick-to-prick (fresh peach), n (%)	29/32 (90.6)
f95-specific IgE and total IgE	
f95 sIgE, kUA/L, median [IQR]	3.06 [0.77–11.74]
Total IgE above age-adjusted normal range, n (%)	36/38 (94.7)
Molecular sensitization profile (CRD)	
rPru p 3 (nsLTP) positive, n (%)	32/38 (84.2)
rPru p 3 sIgE, kUA/L, median [IQR]	1.215 [0.642–13]
rPru p 7 (GRP) positive, n (%)	4/38 (10.5)
rPru p 1 (PR-10) positive, n (%)	3/38 (7.8)
rPru p 4 (profilin) positive, n (%)	1/38 (2.0)
Sensitization pattern	
Single component sensitization, n (%)	33/38 (86.8)
Multiple components sensitization, n (%)	5/38 (13.2)

**Table 4:** Detailed profile of patients with multi-component peach sensitization.

Patients	Age	Total IgE	F95 sIgE	F419 rPru p1 (PR10)	F420 rPru p3 (LTP)	F421 rPru p4	F454 rPru p7	Family atopy	Urticaria	Oral allergy syndrome	Angioedema	Vomiting	Cough	Rhinitis	Bronchospasm	Anaphylactic shock	Hospitalization	Severity class
1	34	3248.5	8.35	23.1	1.25	0	0	+	+	–	–	–	–	+	–	–	–	2
2	11	384.2	0.38	0.01	0.28	0	7.14	+	–	–	+	–	–	+	+	–	–	3
3	15	1245.8	22.6	0.01	16.5	0.02	0.73	+	+	–	+	–	–	–	–	–	+	3
4	12	4306.3	52.9	0.06	80.3	0.06	0.22	+	+	–	+	–	–	+	+	–	+	3
5	65	149.4	0.15	0	0.04	1.4	2.16	–	+	–	–	–	–	–	–	–	–	2

**Figure 4:**
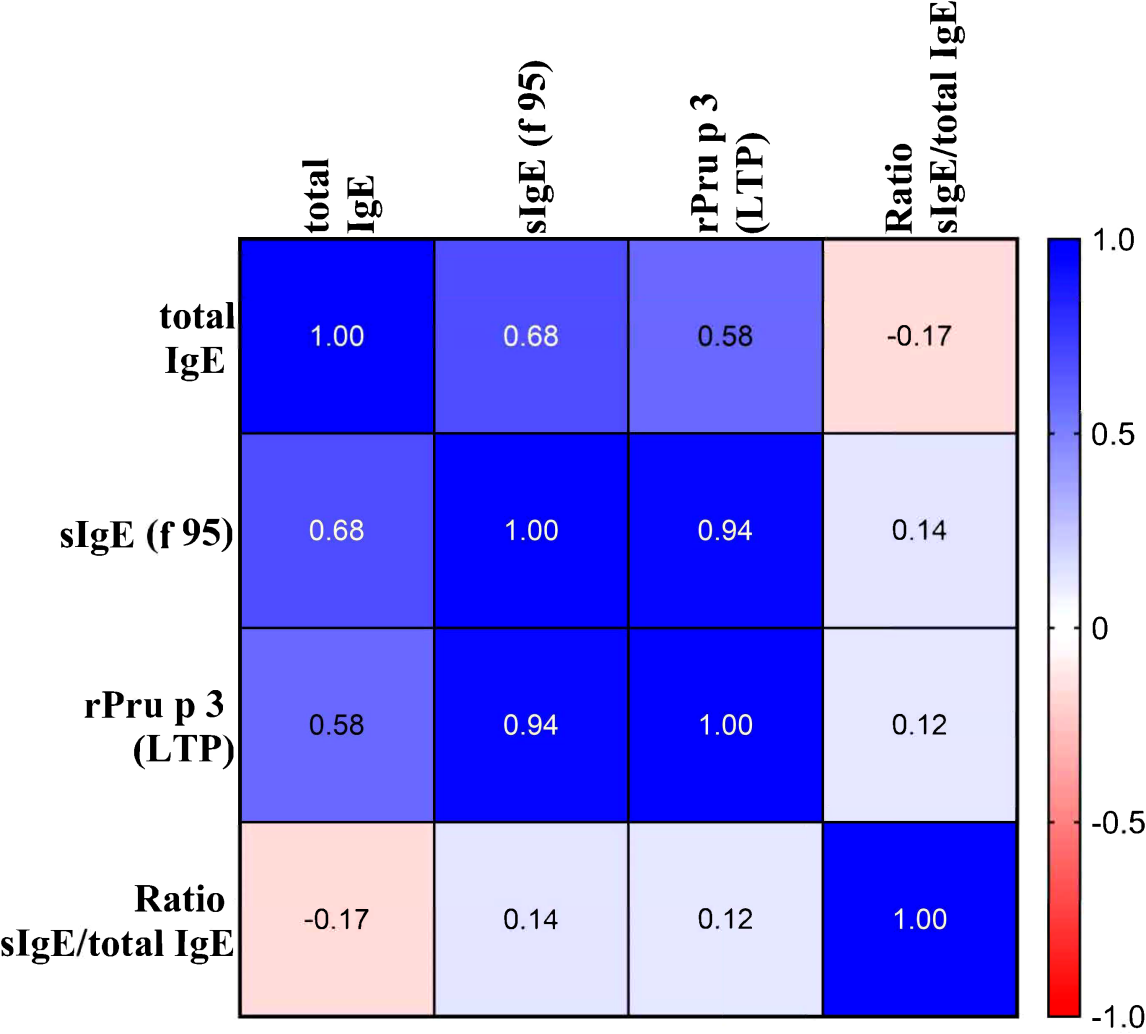
Correlation heatmap of total IgE, specific IgE, and derived ratio.

Among peach-sensitized patients (*n* = 38), sIgE to f95 showed a strong correlation with rPru p 3 (ρ = 0.942, *p* < 0.001), confirmed by linear regression (R² = 0.886, *p* < 0.001) (**Figure 3E**). tIgE also correlated with rPru p 3 (**Figure 4**), albeit moderately (ρ = 0.58, R² = 0.339, *p* < 0.001) (**Figure 3C**).

ROC analysis showed poor discrimination for tIgE (AUC = 0.5; cut-off = 259.4 kU/L: 70.6% sensitivity, 50% specificity) (**Figure 3F**) but better performance for sIgE to f95 (AUC = 0.698; cut-off 0.23 kUA/L: 100% sensitivity, 66.7% specificity) (**Figure 3D**).

Multivariable analysis showed a strong positive association between rPru p 3 and sIgE to f95 levels (β = 1.020, *p* < 0.001), with no significant correlation with tIgE (*p* = 0.141) or age (*p* = 0.564). The model explained 89.5% of the variance in rPru p 3 (R² = 0.895, *p* < 0.001), indicating that rPru p 3 titers above the normal range are primarily driven by peach sIgE, independent of tIgE concentrations or age (**Table 5**).

**Table 5:** Associated factors to r Pru p3 levels by multivariable analysis.

Predictor	β coefficient	T test	p-value	95% CI for β coefficient		Partial correlation
				Inf	Sup	
Age	-0.030	-0.523	0.604	-0.200	0.118	-0.027
sIgE/tIgE	-0.039	-0.642	0.526	-9.437	4.912	0.125
tIgE	-0.134	-1.625	0.114	-0.004	0.000	0.578
sIgE	1.039	12.681	<0.001	1.150	1.589	0.942

Dependent variable: r Pru p3.

### 3.5 Cross-reactivity patterns of peach-sensitized patients

Cross-reactivity testing was conducted in 31 patients, only when clinical symptoms or history were suggestive, or when SPT results were positive and available. Profiles were distributed as follows: food allergens only (*n* = 15, 48.4%), respiratory allergens only (*n* = 7, 22.6%), and both food and respiratory allergens (*n* = 6, 19.4%). Among peach-sensitized patients, cross-reactivity with apple was noted, in 8 cases (88.9%), evidenced by positive apple LTP (rMal d 3), indicating a relatively common LTP syndrome pattern. Respiratory allergen cross-reactivity was observed in 13 patients (41.9%), involving HDMs (*n* = 11, 91.7%), olive (*n* = 5, 62.5%), grass (*n* = 5, 71.4%), cypress (*n* = 2, 40%), and birch (*n* = 2, 100%).

The most commonly positive food and respiratory allergens are summarized in **Table 6**.

**Table 6:** Cross-reactivity patterns in peach-sensitized patients.

Category	Allergen	Positive/Tested	Percentage (%)	Median sIgE (kUA/L) [IQR]
Food allergens	Apple	10/10	100.0	11.03 (6.73-11.95)
	Hazelnut	5/6	83.3	0.38 (0.17-7.08)
	Peanut	5/5	100.0	23.20 (3.53-25.00)
	Apricot	4/4	100.0	1.49 (0.49-3.94)
	Walnut	4/4	100.0	7.81 (3.93-9.10)
	Pistachio	2/3	66.7	2.06 (1.05-2.73)
	Pear	2/2	100.0	9.10 (0.39-17.90)
Respiratory allergens	House dust mites D1	11/12	91.7	0.21 (0.03-2.30)
	Olive pollen	5/8	62.5	4.72 (0.07-31.82)
	Grass pollen	5/7	71.4	–
	Cypress pollen	2/5	40.0	0.03 (0.02-1.40)
	Birch pollen	2/2	100.0	67.25 (51.05-83.67)
Cross-reactivity patterns (*n* = 31) ^*^	Food only	15/31	48.4	–
	Respiratory only	7/31	22.6	–
	Combined food + respiratory	6/31	19.4	–
	No cross-reactivity	3/31	9.7	–

IQR, Interquartile Range (25th-75th percentile).

^*^Cross-reactivity was assessed in 31 peach-sensitized patients when clinically indicated.

**Figure 5:**
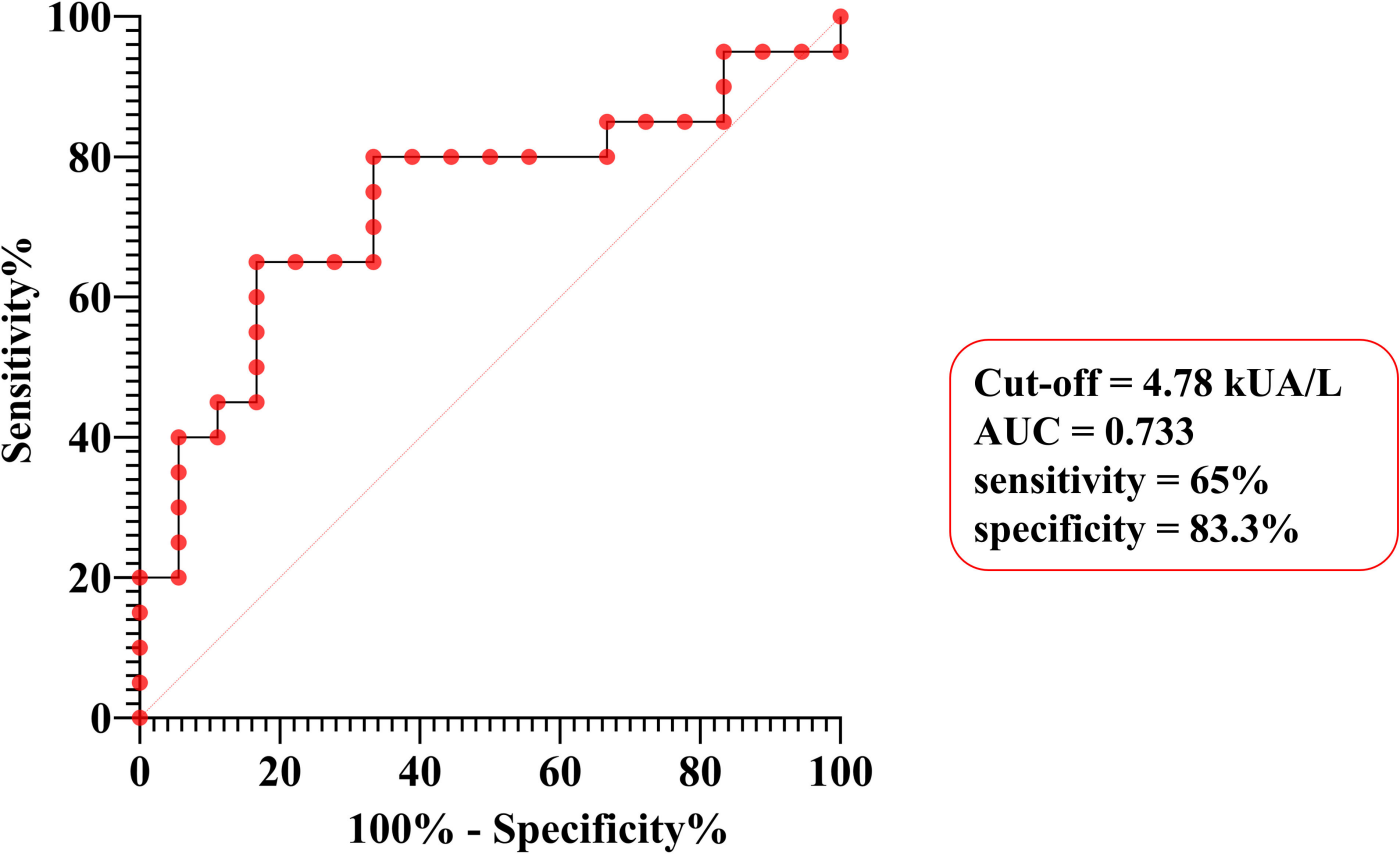
ROC curve analysis for predicting polysensitization using peach-specific IgE levels.

Among 31 patients evaluated for suspected clinical cross-reactivity to other food or respiratory allergens, 20 (52.6%) were classified as polysensitized based on sensitization to three or more different allergens. It is important to note that polysensitization in this context reflects serological reactivity and does not necessarily indicate confirmed clinical allergy to all identified allergens, as systematic oral food challenges were not performed for all cross-reactive foods. Polysensitization was not associated with age (*p* = 0.633), with median ages of 10.5 [7–15] and 9.5 [6–14] years in polysensitized and mono- or bi-sensitized patients, respectively. No statistically significant associations were observed between polysensitization and clinical severity, anaphylactic shock, or hospitalization (all *p*-values > 0.05). Notably, polysensitization was significantly correlated with peach sIgE levels (*p* = 0.013) but showed no correlation with tIgE and rPru p 3 levels (*p* = 0.515 and *p* = 0.082, respectively). ROC analysis demonstrated good discriminative performance (AUC = 0.733) with an optimal cut-off of 4.78 kUA/L for predicting polysensitization, yielding 65% sensitivity and 83.3% specificity (**Figure 5**).

### 3.6 Clinical severity and predictive factors

According to the oFASS-3 classification, reactions were Grade 1 in 5.4%, Grade 2 in 51.4%, and Grade 3 in 43.2%. No significant associations were found between reaction severity and demographic variables or any biomarkers including tIgE, sIgE to f95, rPru p 3, normalized ratios (rPru p 3/sIgE; sIgE/tIgE; rPru p 3/tIgE) and molecular allergens (rPru p 1, rPru p 3, rPru p 4, and rPru p 7) (all *p*-values > 0.05). None predicted anaphylactic shock or hospitalization. No significant association between polysensitized patients and clinical reaction severity according to oFASS-3 classification (*p* = 0.833). The 5 patients with multiple component co-sensitization did not display any distinctive clinical features or specific cross-reactivity patterns with other allergens compared to those with single component sensitization.

## 4 Discussion

This study represents the first comprehensive molecular characterization of peach allergy in the Tunisian population, providing crucial insights into sensitization patterns and clinical manifestations within the North African Mediterranean context. Our findings highlight specific epidemiological and molecular characteristics of peach allergy that both converge with and diverge from previously reported European and Asian data. These insights expand the global understanding of the heterogeneous nature of peach allergy and provide novel contributions to molecular allergology, particularly in resource-limited settings.

In Tunisia, the use of molecular allergology remains recent and is not yet systematically implemented, which partly accounts for the limited number of documented food allergy cases (14), with the notable exception of cow’s milk allergy (15). Against this background, our study comprising 49 patients with suspected peach allergy represents a substantial sample for this setting and allows meaningful comparison with international data. While some studies on *Rosaceae* allergy in children report smaller series (16), other studies from Northern Mediterranean regions investigating the biological features of peach allergy have reported larger cohorts (17, 18).

Among 49 patients with suspected peach allergy, 77.5% tested positive for sIgE to peach extract, a proportion slightly lower yet comparable to the 93% reported in a French study (19). Notably, 11 patients with negative f95-sIgE (<0.1 kUA/L) were classified as allergic based on consistent clinical history and compatible symptoms, prompting further allergological evaluation. Several mechanisms may account for the absence of detectable serum sIgE without implying misdiagnosis. In pediatric populations, particularly in Mediterranean regions, early-stage or low-level sensitization to LTPs may not be reflected in circulating sIgE despite tissue-level allergen-IgE, so clinical symptoms can precede measurable serum IgE, as reported for Pru p 3 and other plant-derived allergens (20, 21). SPT, available for part of this subgroup, was positive in some cases, supporting IgE-mediated sensitization despite negative serum results, consistent with documented discordance between SPT and sIgE in food allergy and reflects differences in the biological compartments assessed rather than test inaccuracy (22). Furthermore, extract-based assays using peach whole extract (f95) show reduced sensitivity for LTP sensitization, compared with labile components, particularly in monosensitized or early-stage patients, whereas CRD provide more accurate detection (9, 23). In our study, molecular allergen testing was performed only in patients with positive f95 results, potentially missing clinically allergic patients with negative f95. These observations underscore the value of a case-by-case application of CRD to complement conventional testing in suspected peach allergy. Accordingly, low-level sensitization (sIgE 0.1–0.35 kUA/L) was included for analytical purposes but was not systematically equated with clinically confirmed peach allergy, particularly in the absence of oral food challenge, and was interpreted with caution in light of clinical history and complementary diagnostic findings.

Importantly, our sIgE-positive patients were significantly younger than the sIgE-negative ones (*p* = 0.021), consistent with Mediterranean pediatric studies showing that early-life exposure to LTP is often associated with earlier onset of clinically relevant sensitization (24).

Our study demonstrates clear age-related patterns in peach allergy, with confirmed peach-allergic patients being significantly younger than non-sensitized individuals (*p* = 0.021). This observation is supported by recent data from southern Tunisia, where peach accounts for 26.8% of clinically diagnosed fruit allergies, despite earlier studies not incorporating molecular diagnostics (25). The pediatric predominance (65.3%) aligns with established epidemiological evidence indicating that IgE-mediated food allergies, particularly fruit allergies, occur predominantly in childhood (20, 26), and with Mediterranean regions, reflecting early LTP sensitization driven by dietary exposure (27). In contrast, Northern European populations typically develop peach allergy later, as documented in the *EuroPrevall* study (28), a pattern that may be influenced by multiple factors, notably differences in molecular sensitization profiles. The median age at first documented allergic reaction reported in our study group was 5.5 years, aligning with Mediterranean pediatric patterns and consistent with Spanish data reporting onset ages between 7.4 and 12 ± 7 years (26, 29). The significant age difference reinforces the importance of early diagnosis in pediatric patients.

The observed sex distribution revealed a male predominance among peach-allergic patients, consistent with age-dependent disparities in IgE-mediated food allergies, which are more frequent in boys during childhood and shift toward female predominance after puberty (30, 31). The *EuroPrevall* study, examining European hospital-based cohorts with mean ages around 28 years, suggested female predominance in adults, supporting this age-related pattern (28). The male predominance in our cohort likely reflects its pediatric composition and may be influenced by broader environmental factors specific to the Tunisian context. Male children may exhibit enhanced Th2-mediated immune responses and increased susceptibility to atopic sensitization during early childhood (32). Nevertheless, larger studies are needed to confirm whether this pattern reflects a true feature in North Africa or merely reflects selection bias.

Our clinical spectrum demonstrated remarkable heterogeneity, with cutaneous manifestations predominating, consistent with Spanish pediatric studies reporting urticaria in 63% of patients (26). Respiratory involvement was observed in 52.6% of our patients, in line with Mediterranean studies. *Cuesta-Herranz* et al. (29) similarly reported allergic respiratory disease in 86% of peach-allergic patients, suggesting that peach allergy may be associated with bronchial hyperreactivity. The respiratory manifestations likely reflect both the local inflammatory response to peach allergens and potential cross-reactivity with respiratory allergens. OAS was documented in 32.4% of our patients, consistent with the 36.7% reported in a Portuguese study (33).

Anaphylactic shock was documented in 10.5% of cases, highlighting a significant burden of severe, life-threatening reactions within our study group. This aligns with reports from Mediterranean populations, where systemic reactions to peach are frequently observed. *Pastorello* et al. (18) reported anaphylaxis in 16% of Italian adults, while *Boyano-Martínez* et al. (26) described severe multisystem reactions in 26% of Spanish children—both exceeding the rate observed in our group. In contrast, lower frequencies of anaphylaxis have been reported in Central and Northern European countries (9), where peach allergy is more commonly linked to pollen-food allergy syndrome (PFAS) and typically presents with milder symptoms. Our findings are broadly consistent with the Mediterranean pattern of more severe, LTP-mediated allergic responses (34). The comparatively lower rate of anaphylaxis observed in our Tunisian cohort may result from multiple, non-exclusive factors. These include a milder regional phenotype, as well as strict avoidance behaviors—widely adopted in Tunisia as a common reflex following allergic reactions—driven by limited exposure to peach, whether through consumption of peach-flavored products or indirect skin contact. In addition, incidental low-level exposures during unsupervised consumption may contribute to partial oral desensitization. Such behavioral patterns and exposure histories could underlie the lower anaphylaxis rates observed and warrant further investigation to better define their role in modulating clinical severity among peach-allergic individuals.

Peach-allergic patients exhibited significantly higher tIgE levels compared to non-sensitized individuals (*p* = 0.002), with 94.7% presenting values beyond the established normal thresholds. The proportion of individuals with tIgE beyond the established normal thresholds in our study exceeds that typically observed in food-allergic populations (60–85%) (35, 36). *Li* et al. (37) reported comparable median tIgE levels in patients with oral allergy syndrome and systemic reactions, but did not specify the number of patients with values above the normal range. This likely reflects the influence of the Mediterranean climate on enhanced atopic sensitization, potential referral bias favoring patients with more complex allergic phenotypes, and the predominantly pediatric composition of our study population, in which children frequently display more pronounced atopic activity. Notably, it has been reported that approximately 20% of allergic patients present with normal tIgE levels (38, 39), while non-atopic patients may exhibit tIgE levels that exceed the established normal threshold (40, 41), highlighting the limited diagnostic value of tIgE. Its clinical utility is therefore more accurately interpreted alongside sIgE measurements. In our study, we observed a moderate but significant correlation between tIgE and peach-sIgE levels (ρ = 0.534, *p* < 0.001), supporting the role of tIgE as an adjunctive marker—an observation further reinforced by ROC analysis. This finding has prompted consideration of sIgE/tIgE ratios to enhance diagnostic performance. In our study, this ratio was significantly higher in patients positive for sIgE to f95 compared to those who were f95-negative (*p* < 0.001). However, neither univariate nor multivariate analyses demonstrated a significant correlation between the sIgE/tIgE ratio and sIgE levels, suggesting that while the ratio may aid in identifying sensitization status, it does not directly correlate with sIgE levels. These findings are in line with those of *Mehl* et al., who reported that food-sIgE/tIgE ratios for diagnosing symptomatic food allergy to cow’s milk, hen’s egg, wheat, or soy yielded results similar to those obtained with food-sIgE alone (42). In a Tunisian study of shrimp-sensitized patients (14), sIgE/tIgE ratio, was significantly correlated with both shrimp and HDMs sensitization. Although the sIgE/tIgE ratio did not correlate with symptom severity in that cohort, previous research suggests that this ratio is more accurate than sIgE alone in predicting oral food challenge outcomes (43–45). Due to its lack of allergen specificity, tIgE should not be used as a standalone diagnostic tool, in line with international guidelines (46).

Among our North African patients, sensitization to Pru p 3 was predominant (84.2%), confirming that the Mediterranean LTP profile extends into this population. Regarding age, the early onset observed in our study group supports primary sensitization to thermostable food allergen Pru p 3, in contrast to the pollen–food allergy syndrome, where respiratory sensitization typically precedes food allergy. In Mediterranean regions, peach allergy is typically driven by LTP sensitization, particularly to Pru p 3, which has been identified as the main trigger of systemic reactions. This finding is consistent with previous studies from Spain and Italy, which reported Pru p 3 sensitization rates of approximately 62% and 42.5%, respectively, and highlighted its strong association with severe clinical outcomes (17, 18). In Tunisia, rPru p 3 emerged as the central allergenic component driving peach sensitization, as f95 showed a robust correlation with rPru p 3 (*p* < 0.001), confirming its predominant role. This finding aligns with *Balsells-Vives* et al. (13), who reported that the Pru p 3/f95 ratio exceeded 1 across both low- and high-level rPru p 3 groups, indicating sensitization to peach is primarily driven by this stable LTP. The moderate AUC observed (0.698) reflects the inherent complexity of using whole allergen extracts to predict responses to individual molecular components, consistent with *Li* et al. (37). Nevertheless, the high sensitivity (100%) ensures reliable screening for clinically relevant Pru p 3 sensitization. The cut-off of 0.23 kUA/L falls within the low-level range described by *Balsells-Vives* et al. (13), where approximately 45% of patients with low Pru p 3 levels exhibited clinically relevant reactions. Notably, when peach-sIgE is below this threshold, molecular LTP testing may have limited utility, and other allergen families should be considered in severe cases. These results validate peach-sIgE as an effective screening tool in Tunisia, while highlighting the added value of CRD for precise characterization of LTP syndrome. The moderate specificity (66.7%) likely reflects cross-reactivity or minor peach allergens, consistent with the Mediterranean phenotype predominantly driven by heat-stable, digestion-resistant LTPs. As emphasized by *Pascal* et al. (47), the ratio of component-specific to whole-extract IgE can help determine the relative contribution of individual allergenic components, particularly when major recognized components are negative despite positive whole-extract IgE results.

In contrast, sensitization to recombinant Pru p 1 was observed in only three patients, supporting the well-established North–South European gradient in peach allergen profiles (48, 49). Among these three patients, one was a 5-year-old child who developed severe reactions, including angioedema and anaphylactic shock. Biological testing revealed broad polysensitization, with all PR-10 molecular allergens exceeding 100 kUA/L, while LTP components were negative, excluding LTP syndrome. The severe reaction was attributed to strong birch pollen sensitization and to α-Gal, explaining the concomitant beef allergy, together with cow’s milk protein allergy. Rosaceae syndrome was confirmed by recombinant PR-10 positivity. Notably, the child had lived in Sweden during the first four years of life, underscoring the role of early birch exposure in shaping sensitization patterns. The low prevalence of IgE reactivity to Pru p 1 (7.8%) in our series is consistent with geographic trends and mirrors Spanish cohorts, where IgE binding to rBet v 1 was reported in only 7% of peach-allergic patients (17). These findings support the concept that Pru p 1 sensitization is predominantly linked to birch pollen–food allergy syndrome, which is uncommon in Mediterranean regions (9). Northern European populations frequently exhibit sensitization to the labile PR-10 protein Pru p 1 (49), whereas Southern and Mediterranean populations—including North Africans, as demonstrated here—are predominantly sensitized to the stable LTP allergen Pru p 3 (8, 50, 51).

Overall, Pru p 7 sensitization was observed in 10.5% of our patients, including one case of isolated reactivity and three cases of concomitant rPru p 3-positivity, highlighting the emerging clinical relevance of this allergen. Spanish pediatric studies reported 16.3% sensitization rates, with significant associations to severe reactions and anaphylaxis (27). Even higher rates have been documented in other populations: *Klingebiel* et al. (10) reported Pru p 7 sensitization in 54% of patients with suspected peach allergy and 62% of those with confirmed peach allergy in southern France. In a Japanese study, 65% of fruit-allergic patients—whose reactions were not attributable to PR-10, LTP, or profilin components—were sensitized to GRP (52). In our study, IgE from four patients bound to rPru p 7, but three also showed IgE reactivity to rPru p 3, leaving only one patient with isolated Pru p 7 sensitization. This 11-year-old boy initially presented with respiratory manifestations—including bronchospasm, rhinitis, and cough, sometimes triggered even by peach fragrance—along with urticaria, reflecting a relatively severe clinical profile. Significant exposure to cypress pollen in northern Tunisia provides a plausible explanation for the observed Pru p 7 sensitization rates. Cypress plantations in this region are widespread and are known to be the dominant species used as windbreaks, accounting for 57% of these plantings (53). This observation supports the concept of “geographic allergenic fingerprints,” where local pollen exposure influences the molecular profiles of food allergies within populations. The mechanistic basis for this association is well established, as Pru p 7 shares structural homology with Cup a 7, supporting cross-reactivity within the Cupressaceae family (54). It is uncertain whether isolated sensitization to Pru p 7 alone accounts for the clinical severity observed in our patient. However, Pru p 7 has recently been confirmed as a significant risk factor for severe peach allergy in both European and Japanese populations, with sensitization correlating strongly with clinical severity and anaphylaxis (10, 28). In addition, it is well documented that sensitization to multiple allergenic components is commonly associated with increased disease severity, as observed in peanut allergy with Ara h 1, Ara h 2 and Ara h (55–57). These findings underscore the need for larger cohorts to better define the role of Pru p 7 sensitization—whether isolated or combined with other molecular allergens—in clinical outcomes.

In our study, rPru p 4 sensitization was detected in only one patient, who also showed IgE reactivity to rPru p 7. This low frequency aligns with previous reports, as Pru p 4—a heat-labile profilin and pan-allergen—is typically associated with mild OAS rather than systemic reactions (9). Sensitization to Pru p 4 often reflects cross-reactivity with pollen profilins (Bet v 2) and generally predicts non-systemic clinical manifestations (9, 17). These observations further support the predominant role of Pru p 3 and the clinical relevance of Pru p 7 in peach allergy.

In both univariate and multivariable analyses, no significant associations were found between reaction severity (oFASS-3) and either tIgE, sIgE to whole peach extract, or sensitization to molecular components such as Pru p 3 or Pru p 1. This suggests potential population-specific variability in clinical expression. Notably, our findings parallel those of *Novembre* et al. (58), who reported no correlation between rPru p 3 levels and severity in 44 peach-allergic children. The predominance of pediatric cases (64.5%) in our study group is also comparable to Spanish pediatric series by *Vílchez-Sánchez* et al. (27), supporting the possibility of age-related differences in biomarker–severity relationships. By contrast, adult studies have yielded opposite results. *Uasuf* et al. (51) demonstrated a strong correlation between Pru p 3-specific IgE levels and reaction severity in southern Italian adults (p < 0.001). Such discrepancies may reflect age-related immunopathophysiological differences, geographical variation in sensitization patterns, or methodological differences in severity assessment. Supporting this complexity, the European-Japanese study by *Kallen* et al. (28) identified Pru p 7 and age at allergy onset as predictors of severity, underscoring the multifactorial nature of biomarker–severity associations in peach allergy.

In our study, 67.7% of tested patients demonstrated sensitization to co-occurring allergens, with food allergens exclusively (48.4%), respiratory allergens alone (22.6%), and combined patterns (19.4%). This pattern supports the concept of LTP syndrome, reflecting the high sequence homology (62–81%) between Pru p 3 and homologous LTPs in Rosaceae fruits (17). Respiratory cross-reactivity was detected in 41.9% of patients, mainly to HDMs (91.7%), olive pollen (62.5%), and grass pollen (71.4%), consistent with typical Mediterranean exposures. In contrast, northern European cohorts more frequently show birch pollen–driven cross-reactivity via Bet v 1 homologs (18). The rarity of birch sensitization in our cohort (*n* = 2) further highlights the predominance of LTP-driven over PR-10–mediated profiles in Mediterranean populations.

Polysensitization affected 52.6% of our patients, consistent with the established framework of LTP syndrome, whereby Pru p 3 sensitization often extends to taxonomically unrelated plant foods containing homologous LTPs. In European cohorts, 91.5% of Spanish peach-allergic patients exhibited LTP syndrome, defined by sensitization to peach and at least one additional LTP-containing plant food (34). This discrepancy likely reflects differences in study populations, as the cited cohort included only adults with confirmed Pru p 3 sensitization. In our study population, polysensitization was not associated with tIgE or IgE levels to Pru p 3, contrasting with previous reports linking polysensitization to tIgE levels above the reference range (R^2^ = 0.465, *p* ≤ 0.001), likely reflecting enhanced IL-4 responses in activated peripheral blood mononuclear cells (59, 60). Notably, polysensitization was significantly associated with f95 levels (*p* = 0.013), with ROC analysis identifying a cutoff of 4.78 kUA/L predictive of polysensitization, offering a practical tool for identifying patients requiring extended cross-reactivity evaluation and facilitating personalized management in resource-limited settings. Our results did not show an association between polysensitization and reaction severity. In contrast, the Spanish adult cohort reported that anaphylaxis was more frequent in patients sensitized to ≥3 plant food groups, suggesting that extensive LTP sensitization may increase the risk of severe reactions (*p* = 0.04) (34).

The universal avoidance strategy, adopted by 97.3% of our patients, may be overly restrictive, as it prevents reactions but does not address underlying immunological dysfunction and may perpetuate Th2-driven inflammatory responses. *Boyano-Martínez* et al. demonstrated that over 90% of Spanish children safely tolerated carefully peeled and rinsed peach pulp, indicating that complete avoidance may not be required (26), reflecting the predominant localization of Pru p 3 in the peel. This supports the potential for individualized dietary management guided by molecular sensitization profiles (61). Contemporary evidence increasingly supports oral immunotherapy and sublingual immunotherapy as transformative alternatives, providing cross-protection against other LTP-containing foods and addressing the broader LTP syndrome (62). Individualized risk assessment remains essential, considering reaction severity, cofactors, concurrent asthma, and psychosocial readiness to guide patient selection and protocol design.

This study has some limitations. The small sample size reduced the statistical power of the analyses, particularly for exploring biomarker associations. Although conducted in a single center, this laboratory serves as the national reference for molecular allergy testing in Tunisia, receiving samples from both public and private healthcare sectors across the country. The limited number of cases primarily reflects the still-limited referral of patients for molecular testing, a situation that is gradually improving as clinician awareness increases. Importantly, as a retrospective analysis, the study also exposes gaps in patient referral, testing practices, and diagnostic pathways, highlighting systemic areas where improvements could enhance allergy care. A notable limitation of our study is that OFC was not performed in any patient. Although the diagnosis was supported by clinical history, documented allergic reactions, and molecular testing, the absence of OFC precludes definitive confirmation of clinical allergy and should be considered when interpreting the prevalence and severity of reactions in this cohort.

By examining real-world data nationwide, these findings provide actionable insights to guide diagnosis, risk stratification, patient counseling, and potential immunotherapy strategies.

These limitations underscore the need for larger, prospectively designed studies with longitudinal follow-up to assess disease severity and molecular profiles, including sensitization patterns to key peach allergens such as Pru p 3 and Pru p 7. Consequently, our findings highlight the importance of cautious, individualized patient assessment, with OFC incorporated in future studies to validate and refine molecular and serological diagnostic approaches. Such prospective approaches will confirm and extend our findings, clarify the natural history of peach allergy, and support the development of more effective, evidence-based diagnostic and management strategies.

## 5 Conclusion

This study provides the first comprehensive molecular characterization of peach allergy in Tunisia, revealing a predominance of Pru p 3 sensitization and the emerging relevance of Pru p 7, likely influenced by local environmental exposures such as cypress pollen. While broadly consistent with the Mediterranean pattern of LTP-driven reactions, the Tunisian population appears to exhibit a milder clinical phenotype, with earlier age at onset and distinct polysensitization profiles. Whole-extract sIgE and CRD offer complementary insights for identifying at-risk patients, whereas total IgE and sIgE/tIgE ratios provide useful adjunctive information without directly predicting sIgE levels. Polysensitization correlated with whole-peach extract sIgE levels, providing a practical marker for cross-reactivity evaluation, whereas molecular sensitization patterns were not associated with reaction severity. Importantly, the high sensitivity of peach-sIgE (100%) ensures reliable screening for clinically relevant Pru p 3 sensitization, with a cut-off of 0.23 kUA/L providing a practical threshold to identify at-risk patients. These findings validate peach-sIgE as an effective screening tool in Tunisia, while highlighting the added value of CRD for precise characterization of LTP syndrome. This study provides novel, region-specific evidence with direct implications for allergy diagnosis, patient counseling, and potential immunotherapy strategies. Despite limitations in sample size, retrospective design, and variability in patient presentation, it establishes a molecular framework for evidence-based management of peach allergy in Tunisia, highlights geographic differences in allergy patterns, and supports region-specific clinical strategies in resource-limited settings. Collectively, these findings underscore the value of integrating molecular diagnostics with clinical care and lay the groundwork for future prospective multicenter studies to validate and expand upon these results.

## Data Availability

The datasets presented in this study can be found in online repositories. The names of the repository/repositories and accession number(s) can be found in the article/**Supplementary Material**.
