# Supplementary material for: The molecular landscape of peach allergy in Tunisia: new insights
Source: Front Immunol. 2026 Mar 3;17:1696481. doi: 10.3389/fimmu.2026.1696481 (PMC12992302; doi:10.3389/fimmu.2026.1696481)
Supplement: Supplementary file 1 [file Table1.docx]

ALLERGOLOGY DEPARTMENT –PASTEUR INSTITUT OF TUNIS

**CLINICAL AND BIOLOGICAL DATA SHEET :Molecular Profiles of Peach Sensitization in Tunisia**

# 1. GENERAL INFORMATION

Initials (Last name / First name): ____ / ____
Sex: ☐ M / ☐ F
Date of birth: //____
Age at sampling: ____ years ____ months
Date of sampling: //____
Referring institution: __________________________
Region of residence: ☐ Greater Tunis ☐ North ☐ Center ☐ South
Phone (patient/contact): ______________________

# 2. PERSONAL AND FAMILY HISTORY

**2.1. Personal history of atopy**
Personal atopy: ☐ Yes / ☐ No
If yes, specify:
☐ Asthma (onset age: ____ years) – Control: ☐ Good ☐ Partial ☐ Poor
☐ Atopic dermatitis (onset age: ____ years) – Severity: ☐ Mild ☐ Moderate ☐ Severe
☐ Allergic rhinitis (onset age: ____ years) – Type: ☐ Seasonal ☐ Perennial
☐ Allergic conjunctivitis
☐ Drug allergies: __________________________
☐ Others: __________________________

**2.2. Family history of allergy**Family history of allergy: ☐ Yes / ☐ No
If yes, specify:
Father: ☐ Asthma ☐ Food allergy ☐ Rhinitis ☐ AD ☐ Other: ______
Mother: ☐ Asthma ☐ Food allergy ☐ Rhinitis ☐ AD ☐ Other: ______
Siblings: ☐ Asthma ☐ Food allergy ☐ Rhinitis ☐ AD ☐ Other: ______
Grandparents: __________________________

# 3. CLINICAL DATA SPECIFIC TO PEACH ALLERGY

**3.1. Sensitization history**
Age at first reaction to peach: ____ years
Circumstances of first reaction: __________________________
Time to symptom onset after ingestion:
☐ < 5 min ☐ 5–15 min ☐ 15–30 min ☐ 30 min–1h ☐ 1–2h ☐ > 2h
Total number of documented reactions: ____
Clinical course: ☐ Stable ☐ Worsening ☐ Improvement ☐ Variable

**3.2. Exposure characteristics**
Peach form involved:
☐ Fresh raw ☐ Fresh cooked ☐ Canned ☐ Dried
☐ Juice ☐ Compote ☐ Jam ☐ Pastries
☐ Other preparation: ___________
Estimated reactive threshold:
☐ < 1 piece ☐ 1 piece ☐ 1/2 fruit ☐ 1 whole fruit ☐ > 1 fruit ☐ Unknown
Reaction reproducibility: ☐ Systematic ☐ Intermittent ☐ Single occurrence

# 4. CLINICAL MANIFESTATIONS

**4.1. Oral Allergy Syndrome (OAS)**
Presence of OAS: ☐ Yes / ☐ No
If yes:
Exclusive? ☐ Yes / ☐ No
Severity: ☐ Mild ☐ Moderate ☐ Severe
Duration: ☐ < 30 min ☐ 30 min–2h ☐ > 2h

**4.2. Systemic manifestations**
Cutaneous symptoms: ☐ Localized urticaria ☐ Generalized urticaria ☐ Angioedema (location: ______) ☐ Contact eczema ☐ Flush ☐ Generalized pruritus
Respiratory symptoms: ☐ Rhinitis ☐ Conjunctivitis ☐ Cough ☐ Voice loss ☐ Bronchospasm ☐ Dyspnea ☐ Laryngeal edema
Digestive symptoms: ☐ Nausea ☐ Vomiting ☐ Abdominal pain ☐ Diarrhea ☐ Others: ______
Cardiovascular symptoms: ☐ Hypotension ☐ Tachycardia ☐ Malaise ☐ Syncope ☐ Anaphylactic shock

**4.3. Severity classification**
Hospitalization required: ☐ Yes / ☐ No. If yes, duration: ____ hours/days
Emergency treatment received: ☐ Antihistamines ☐ Corticosteroids ☐ Epinephrine ☐ Bronchodilators ☐ Others: ______

# 5. ALLERGOLOGICAL WORKUP

**5.1. Skin tests**
Skin prick tests performed: ☐ Yes / ☐ No – Date: //____
Extracts tested and results (wheal diameter in mm):
Fresh peach (prick-to-prick): ____ mm
Commercial peach extract: ____ mm
Histamine (positive control): ____ mm
Saline (negative control): ____ mm

**5.2. Oral food challenge (OFC)**
OFC performed: ☐ Yes / ☐ No – Date: //____
If yes:
Indication: ☐ Diagnostic ☐ Threshold evaluation ☐ Follow-up ☐ Other: ______
Protocol used: __________________________
Cumulative tolerated dose: ____ g
Outcome: ☐ Positive ☐ Negative ☐ Discontinued
Symptoms observed: __________________________
Treatment required: ☐ None ☐ Antihistamines ☐ Corticosteroids ☐ Epinephrine

# 6. CROSS-REACTIVITY AND CO-SENSITIZATIONS

**6.1. Pollen-food syndrome**
Documented pollen allergy: ☐ Yes / ☐ No
If yes, specify pollen(s):
☐ Birch ☐ Olive ☐ Cypress ☐ Grasses ☐ Others: ______
Associated symptoms: ☐ Rhinitis ☐ Asthma ☐ Conjunctivitis – Period: ______

**6.2. Food cross-reactivity**Other Rosaceae fruits (Apricot, Apple, Pear, Plum, Cherry, Almond)
Options: ☐ Tolerated ☐ OAS ☐ Systemic reaction ☐ Avoided ☐ Never tested
Other fruits: Kiwi, Avocado, Banana, Others: ______
Tree nuts: Hazelnut, Walnut, Others: ______

## 7. MANAGEMENT AND FOLLOW-UP

**7.1. Avoidance measures**
Dietary avoidance recommended: ☐ Total ☐ Partial ☐ None
If partial, specify: ☐ Raw peach only ☐ Depending on tolerance ☐ Other: ______
Extended avoidance to other foods: ☐ Yes / ☐ No. If yes, specify: ______
Compliance: ☐ Excellent ☐ Good ☐ Partial ☐ Poor
 **7.2. Prescribed treatment**
Maintenance therapy: ☐ None ☐ Antihistamines ☐ Inhaled corticosteroids ☐ Others: ______
Emergency treatment: ☐ Oral antihistamines ☐ Oral corticosteroids ☐ Epinephrine auto-injector ☐ Written action plan provided ☐ Others: ______
Patient education provided: ☐ Yes / ☐ No

**7.3. Medical follow-up**
Specialized follow-up planned: ☐ Yes / ☐ No
If yes, specialty(ies): ☐ Allergology ☐ Pulmonology ☐ Pediatrics ☐ Internal Medicine ☐ Other: ______
Follow-up frequency: ☐ 6 months ☐ 1 year ☐ 2 years ☐ Depending on evolution ☐ Other: ______
